# Effects of extract solution from magnesium alloys supplemented with different compositions of rare earth elements on *in vitro* epithelial and osteoblast progenitor cells

**DOI:** 10.3389/fbioe.2023.1138675

**Published:** 2023-05-10

**Authors:** Sheng Nie, Jiakai Chen, Chen Liu, Chenhui Zhou, Jikuang Zhao, Zhepei Wang, Jie Sun, Yi Huang

**Affiliations:** ^1^ Department of Neurosurgery, Ningbo First Hospital, Ningbo University, Ningbo, Zhejiang, China; ^2^ Department of Neurosurgery, The First Affiliated Hospital of Xi'an Jiaotong University, Xi’an, China; ^3^ Ningbo Branch of China Academy of Ordnance Science, Ningbo, Zhejiang, China; ^4^ Key Laboratory of Precision Medicine for Atherosclerotic Diseases of Zhejiang Province, Ningbo, Zhejiang, China

**Keywords:** magnesium alloys, epithelial, osteoblast, progenitor cell, rare earth element

## Abstract

**Background:** Magnesium alloys (Mg-alloys) have gained significant attention in recent years as a potential bioactive material for clinical applications. The incorporation of rare earth elements (REEs) into Mg-alloys has been of particular interest due to their potential to improve both mechanical and biological properties. Although there are diverse results in terms of cytotoxicity and biological effects of REEs, investigating the physiological benefits of Mg-alloys supplemented with REEs will help in the transition from theoretical to practical applications.

**Methods:** In this study, two culture systems were used to evaluate the effects of Mg-alloys containing gadolinium (Gd), dysprosium (Dy), and yttrium (Y): human umbilical vein endothelial cells (HUVEC) and mouse osteoblastic progenitor cells (MC3T3-E1). Different compositions of Mg-alloys were assessed, and the effects of the extract solution on cell proliferation, viability, and specific cell functions were analyzed.

**Results:** Within the range of weight percentages tested, the Mg-REE alloys did not exhibit any significant negative impacts on either cell line. Interestingly, moderate compositions (Mg-1.5Gd-1.5Dy-0.825Y-0.5Zr and Mg-2Gd-2Dy-1.1Y-0.5Zr) demonstrated a tendency to enhance osteoblastic activity and promote the vascularization process in both HUVEC and MC3T3-E1 cell lines.

**Discussion:** The results of this study provide valuable insights into the potential benefits of REE-supplemented Mg-alloys for clinical applications. The observed enhancement in osteoblastic activity and promotion of vascularization processes suggest that optimizing the compositions of REEs in Mg-alloys could lead to the development of novel, more effective bioactive materials. Further investigations are required to understand the underlying mechanisms and to refine the alloy compositions for improved biocompatibility and performance in clinical settings.

## 1 Introduction

Implants developed from metal materials have been widely used in our life since the 1960s (Yang et al., 2017). Materials with advanced biological features were further proposed and investigated since the mid-1980s. As the understanding of human immunity and pathophysiology deepens nowadays, such bio-functional or bioactive materials are still on the frontiers of technological innovation. On top of their original mechanical properties as implants with minimal host interaction with the human body, newer generations of bio-functional materials were manufactured with resorbable or bioactive characteristics. This indicated a huge expansion of the applications of metal-based biomaterials, including some biodegradable alloys such as magnesium alloys and zinc alloys. They are proposed to be able to interact with the host’s innate machinery through biophysical or biochemical products (Zeugolis and Pandit, 2015). This brought the research on biomaterials to a whole new level, with a promising future where they are employed to accelerate healing or regeneration processes through impacting tissue repair or even epigenetic modifications (Hench et al., 2003; Mousa et al., 2016). Different metal-based alloys have been used in various areas, from prosthodontic restoration to joint replacement (Semlitsch, 1987; Wataha, 2002). The features of such alloys, to a large extent, rely on the main structural metal, which shapes the strength, elastic modulus, corrosion performance, color, or even thermal expansion (Wataha, 2002). Magnesium, as an essential element of the human body, was employed in the medical field in a wire form to stop bleeding in 1878 (Huse, 1878). Many advances have been achieved to improve its deliverability, strength as well as ductility for different purposes (Windhagen et al., 2013; Kang et al., 2017). Through alloying other elements into the main magnesium structure, the overall mechanical properties could be even more enhanced in comparison with alloys that were engineering-designed. There is also an enormous impact on biocompatibility. One common group of elements that can be incorporated to generate new Mg alloys are those that already exist in the human body with relatively high levels, for example, calcium, zinc, etc. The proportion of these elements is extensively explored and precisely controlled to maintain adequate corrosion resistance in order to keep the strength of the alloys while allowing them to be efficiently elongated. Meanwhile, in some cases, the unavoidable biodegradation and corrosion of Mg alloys can be helpful in releasing alloying elements locally to assist with biological functions. The introduction of other elements, on the other hand, can limit the corrosion rates of Mg alloys from being too rapid, thus preventing excessive hydrogen accompanying the degradation, which has hampered the clinical acceptance of Mg (Wang et al., 2020). Rare earth elements (REEs) are a group of good examples that contribute greatly to the mechanical, corrosion, and biological properties of Mg alloys. These elements that are undergoing extensive studies include group III metals such as scandium (Sc) and yttrium (Y), also lanthanides such as lanthanum (La), neodymium (Nd), dysprosium (Dy), gadolinium (Gd), to name a few. Despite the limited amount of these REEs naturally in the human body, they surprisingly interact with various types of functional pathways, probably due to their similar ionic radius to that of calcium ions (Aylward and Findlay, 1973; Weng et al., 2021). REEs are therefore contributing to a wide series of biological processes from skeletal development and immune response to even memory retention (Evans, 1990). They also possess other interesting properties that are beyond the major biological roles of calcium, such as anticoagulative properties without a clear mechanism (Beaser et al., 1942; Hirano and Suzuki, 1996).

Gd, Dy, and Y are considered as efficient solid solution strengtheners, grain refiners, and texture modifiers for the Mg matrix (Stanford et al., 2011; Yang et al., 2011; Zhang et al., 2011). The maximum solid solubility of Gd, Dy and Y in *α*-Mg reaches up to 23.5 wt%, 25.3 wt%, and 12.47 wt%, respectively, which enables few second phase formation, inhibiting the galvanic corrosion. Although it has been reported that the solid solubility decreases with decreasing temperature, Gd, Dy, and Y still maintained a high level in *α*-Mg compared with other alloying elements, especially with low contents. It is worth mentioning that even though the human body can accept lower contents of these elements, the biosafety of most of them is to be determined. Their biological effects on the vascular/skeletal system are worth being explored to a more extensive level.

In this study, we constructed a new type of Mg alloy in combination with different amounts of Gd, Dy, and Y (referred to as GDY-Mg alloy in the following texts). The effects of the extracts from these GDY-Mg alloys on the *in vitro* endothelial and osteoblast culture systems were simulated and observed.

## 2 Materials and methods

### 2.1 Production of magnesium alloys of five different compositions

The experimental GDY-Mg alloys were fabricated. Pure Mg (99.9%) was molten in a high-purity graphite crucible under the protection of a mixed gas atmosphere of SF_6_ (1 vol%) and carbon dioxide (CO_2_). Mg-20 wt% Gd master alloy, pure Dy (99.5%), Mg-20 wt% Y master alloy, and Mg-30 wt% Zr master alloy were orderly added to the melt at 720°C in nominal amounts. The melting was held for 30 min and stirred with a graphite rod. The melt was then poured into a steel mold preheated at 300°C. After casting, both the tops and bottoms of the ingots were cut away, and the middle parts were then homogenized at 520°C for 24 h followed by quenching in water. The as-quenched ingots were hot extruded into bars at 400°C with the extrusion ratio of 64:1 at a ram speed of 10 mm s−^1^. T.

The composition of the GDY-Mg alloys measured by inductively coupled plasma atomic emission spectrometry (ICP-AES, Optima 7300DV, PerkinElmer, Waltham, MA, United States) is listed in Table 1.

**TABLE 1:** Composition of five types of alloys verified by inductively coupled plasma atomic emission spectrometry (ICP-AES, Optima 7,300 DV, PerkinElmer, Waltham, MA, United States).

Alloy	Code	Gadolinium (Gd, wt%)	Dysprosium (Dy, wt%)	Yttrium (Y, wt%)	Zirconium (Zr, wt%)	Magnesium (Mg)
Mg-0.5Gd-0.5Dy-0.275Y-0.5Zr	GDY0.5	0.36	0.6	0.14	0.5	Bal
Mg-1Gd-1Dy-0.55Y-0.5Zr	GDY1.0	0.71	1.34	0.27	0.52	Bal
Mg-1.5Gd-1.5Dy-0.825Y-0.5Zr	GDY1.5	1.36	2.48	0.93	0.55	Bal
Mg-2Gd-2Dy-1.1Y-0.5Zr	GDY2.0	1.83	3.48	1.29	0.54	Bal
Mg-2.5Gd-2.5Dy-1.375Y-0.5Zr	GDY2.5	2.32	4.09	1.95	0.52	Bal

### 2.2 Composition of the elements in the magnesium alloy

#### 2.2.1 Preparation of the solution

The samples of alloys were fully immersed in serum-free *α*-MEM medium (Gibco) and endothelial maintenance medium (Sciencell) for 24 h at 37°C ± 0.5 C in an incubator. Solid alloys were discarded. Liquid extracts were centrifuged at 5,000 r/min for 5 min. The supernatants were purified with 0.22 μm filters and kept in sterile conical tubes at 4°C.

### 2.3 Cell culture

To assess the impact of GDY-Mg alloy on the osteogenic potential of osteoblasts, a sub-line (MC3T3-E1, ATCC: CRL-2594) of an osteoblast precursor cell line derived from *Mus musculus* calvaria was used and the cells were purchased from the Shanghai Cell Bank of the Chinese Academy of Sciences (Kodama et al., 1981). This cell line was maintained in non-osteogenic *α*-MEM (Gibco) with 10% FBS (Gibco). Another cell line, human umbilical vein endothelial cells (HUVECs, ATCC: CRL-1730), the cells were purchased from the Shanghai Cell Bank of the Chinese Academy of Sciences., were employed in this study to explore the effects of different compositions of GDY-Mg alloys on promoting the vascularization process. Cells were maintained in a humidified atmosphere with 5% CO_2_ at 37°C ± 0.5 C and passaged at 60%–70% confluency.

### 2.4 Live/dead cell staining

Cells were seeded into 24-well plates in advance till 80% confluency. The media in experimental wells were switched to extraction with 10% FBS, while the media in the control wells were changed to fresh regular media. Cell viability after a 24-h period of incubation with extract solutions was visualized with a double staining kit (Sigma-Aldrich). The staining kit contains acetoxymethyl ester of calcein (Calcein-AM) and propidium iodide (PI) solutions, which stain viable and dead cells, respectively. The eventual concentrations were 2 μmol/L for calcein-AM and 4 μmol/L for PI. Simultaneous monitoring of viable versus dead cells was observed at excitation 490 nm using a fluorescence microscope (Olympus IX71, Japan).

### 2.5 Proliferation assay

The 3-(4,5-dimethylthiazol-2-yl)-2,5-diphenyltetrazolium bromide or MTT assay was used to measure cellular metabolic activity as an indicator of cell proliferation. Cells were seeded into 96-well plates. Media was switched to Extract with 10% FBS when the plates were at 75%–80% confluency, and the Fresh extract with 10% FBS was changed every other day. Cell proliferation activity was measured on Day 1, Day 3, and Day 5 after the extract was introduced. The incubation period with 5 mg/mL MTT was 4 h, and the absorbance was read at 490 nm.

### 2.6 Cytoskeleton staining with phalloidin

Rhodamine phalloidin (Cytoskeleton, Inc.) was used as the fluorescent marker/stabilizer for staining the actin cytoskeleton in fixed cells. Cells were seeded into 12-well format glass plates (Corning) till 80% confluency. The media were switched to extract media supplemented with 10% FBS for 12 h. Then the cultures were fixed with the 4% polyformaldehyde fix solution (Thermo Scientific) for 30 min at 4°C. After rinsing with PBS at room temperature for 30 min and permeabilization with Triton X-100 for 5 min, 300 μL of 100 nM rhodamine phalloidin was added. The incubation system was kept in the dark at room temperature for 30 min. Afterward, the staining was terminated by removing all the incubation liquid and rinsing the plates three times in PBS. Additionally, the nuclei were stained with DAPI (Dojindo) in the dark for 1 min, followed by rinsing three times in PBS. A fluorescent microscope (Olympus IX71, Japan) was used to observe the results with an excitation filter at 535 nm and an emission filter at 585 nm for rhodamine; an excitation filter at 355 nm, and an emission filter at 460 nm for DAPI.

### 2.7 Alkaline phosphatase (ALP) activity

MC3T3-E1 cells were seeded into 12-well plates till they reached about 80% confluency. The following recipe was used to make the initiation media for inducing osteoblastic processes: 100 nM dexamethasone (Gibco), 0.2 mM ascorbic acid (Gibco), and 10 mM *β*-glycerophosphate disodium salt hydrate (Millipore Sigma), diluted with extract supplemented with 10% FBS. The media were changed every 3 days. To avoid degradation of ascorbic acids, fresh media were made when needed and kept wrapped with alumni foil in the dark.

An assay from Beyotime using p-nitrophenyl phosphate (pNPP) was employed to measure alkaline phosphatase activity during the induced osteoblastic process in MC3T3-E1 cultures. The phosphate substrate pNPP will turn yellow when dephosphorylated by ALP (λmax = 405 nm). The amount of total protein was measured with the MiroBCA kit (Thermo Scientific) at 562 nm, and the ALP activity was calculated as:

$$ALP\;activity\;\left(\frac{mmol}{g}protein\right)=\frac{concentration\;of\;ALP\;\left(\frac{mmol}{L}\right)}{Total\;protein\;\left(\frac{g}{L}\right)}\times 100\%$$

All detailed procedures of the abovementioned assays were conducted according to the manufacturer’s instructions unless otherwise specified.

### 2.8 HUVEC tube-formation assay

*In vitro* angiogenesis of HUVECs was evaluated with a tube-formation assay in this study, reflected by the ability of HUVECs to form three-dimensional capillary-like tubular structures when cultured on Matrigel (Corning) (Ko and Lung, 2012).

Matrigel was thawed at 4°C overnight, and its liquid was kept on ice during the plating. Then 50 μL of Matrigel was plated to 96-well plates at a horizontal level that allowed the Matrigel to distribute evenly and incubated for 30 min at 37°C. Cells were detached, dissociated, and re-suspended with extraction supplemented with 10% FBS to the cell suspension and loaded on top of the Matrigel (Au - Francescone et al., 2011). They were observed at 4, 8, and 16 h under the microscope.

### 2.9 Statistical analyses

Shapiro-Wilk normality tests were applied to test distributions of the data. Ordinary one-way analyses of variances (ANOVA) were employed when multiple comparisons among groups were needed, with Tukey’s multiple comparisons test as the *post hoc* test for adjusted *p* values. A *p*-value less than 0.05 was considered statistically significant.

## 3 Results

### 3.1 Composition of five types of GDY-Mg alloys verified by inductively coupled plasma atomic emission spectrometry (ICP-AES, optima 7300 DV, PerkinElmer, waltham, MA, United States)

**FIGURE 1:**
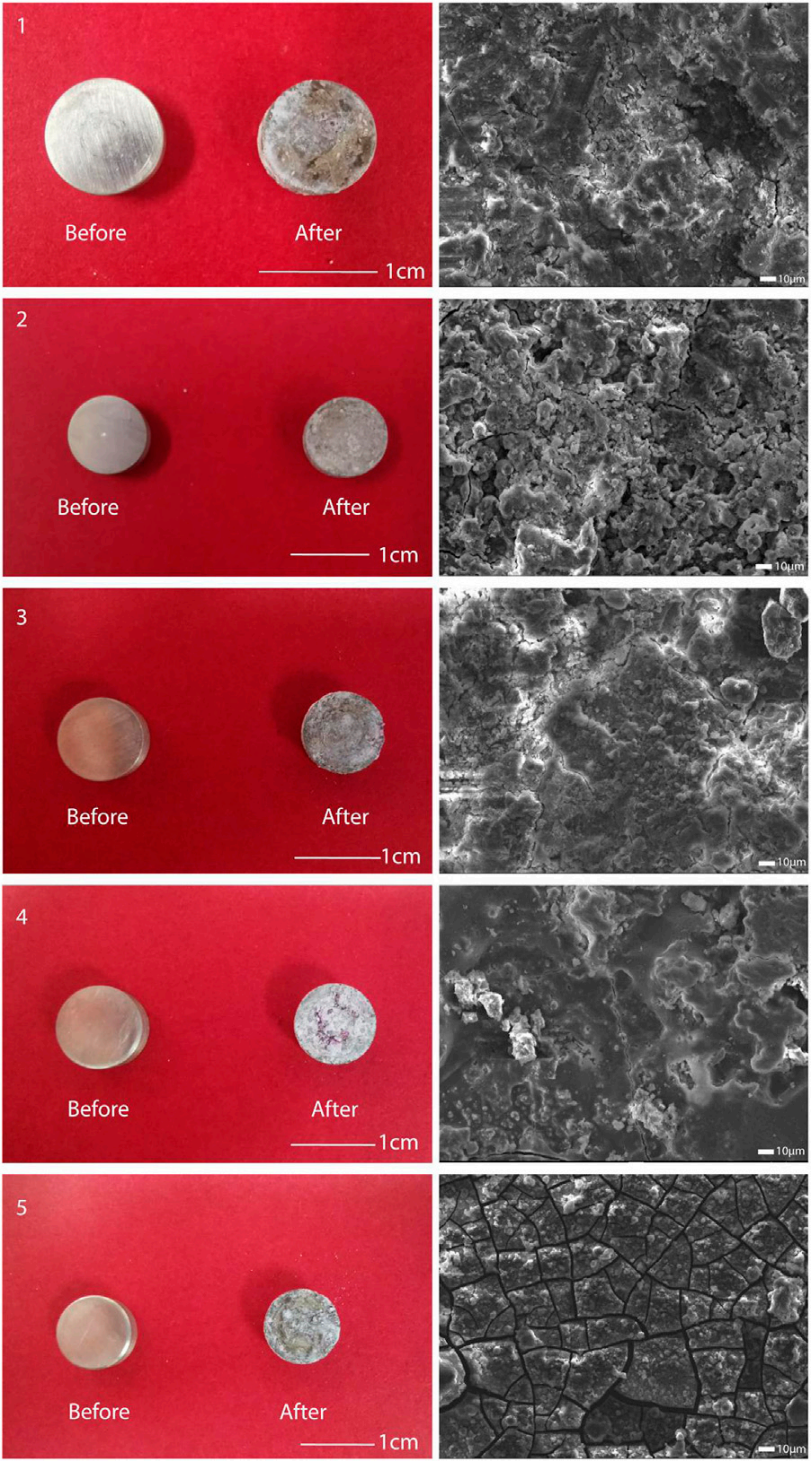
Observation of the surface of five types of alloys. Left: surface conditions of the five alloys before and after immersion; right: surface structure of the five alloys after immersion observed using electron microscopy.

The accurate compositions of five types of GDY were measured and presented in Table 1. Based on the desired weight percentage of Gd, Dy, and Y elements, these five types were coded GDY0.5 to GDY2.5, respectively. The experimental groups that received extracts from each GDY sample were later referred to as Groups 1 through 5, with group 5 containing the highest amount of the three abovementioned REEs. The desired amounts of Gd, Dy, and Y elements were kept as 2:2:1, and 0.5 wt% of zirconium was universally incorporated for grain refinement. The rest metal composition of these alloys was all balanced with magnesium. The percentages of three REEs measured were not precisely the same as expected, but the desired trend was achieved: increased proportions of Gd, Dy, and Y from GDY0.5 to GDY2.5, with a consistent percentage of Zr (Table 1). The surface of the treated material changed before and after immersion of each type of GDY material, and electron microscopy observed that each type of GDY showed a different surface structure after immersion, as shown in Figure 1.

### 3.2 Effects on osteoblastic processes

The effects of the GDY-Mg alloy extract solution on both osteoblast precursor cells (MC3T3-E1) and endothelial cells (HUVECs) were preliminarily investigated from three aspects: cell proliferation rate when treated with extract solution for up to 5 days; cell viability after incubation with the extract solution for 24 h; and thirdly, the potential impacts on their cytoskeleton structures.

**FIGURE 2:**
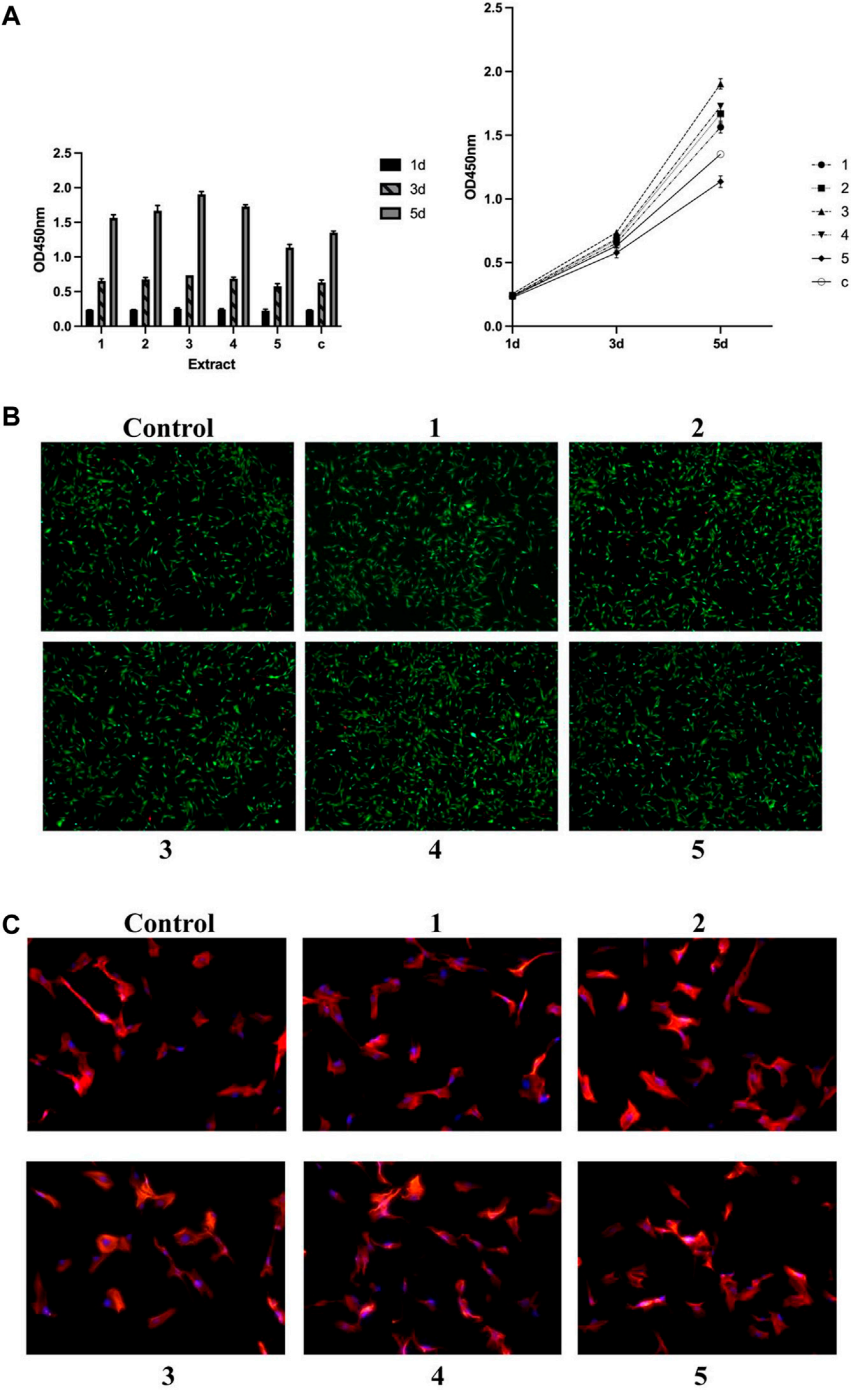
Effects of metal extract solutions from different compositions of GDY-Mg alloys on the viability, proliferation, and cytoskeleton structure of MC3T3-E1 cell culture. **(A)** Cell proliferation levels visualized by group (left) and time point (right). **(B)** Live/dead staining. **(C)** Cytoskeleton staining with phalloidin. 1 to 5 are GDY-Mg alloys from GDY0.5 to GDY2.5, and c is the control group with the plain medium.

The stimulative effects of GDY-Mg alloy extract solution on the proliferation of osteoblast progenitor MC3T3-E1 cells started to vary on day three of treatment, depending on the compositions of the original alloys (Figure 2A). Cells treated with extract of Alloy type three (GDY1.5) proliferated faster than those treated with type 1 (GDY0.5), *p* = 0.0447. Alloy 2, Alloy 3, and Alloy 4 extract all enhanced the proliferation higher than Alloy 5 to a statistically significant level (*p* = 0.0134, 0.0003, and 0.0069, respectively). Alloy 3 seemed to have the strongest stimulative effect that was significantly higher than plain medium (*p* = 0.0101).

Such trends became more obvious when the treatment lasted till day five. Cells with Alloy 3 extract solution proliferated the fastest compared to those treated with lower or higher compositions of GDY-Mg alloys (compared to Alloy 1: *p* < 0.0001; Alloy 2: *p* = 0.0004; Alloy 4: *p* = 0.0051). Alloy 5 extract solution exhibited a negative effect on the proliferation of MC3T3-E1 cells (*p* < 0.0001 compared to Alloy 1, Alloy 2, Alloy 3, and Alloy 4; *p* = 0.0009 compared to the control group).

Viability labeling was done at the 24-h time point as a cross-sectional view of live/dead cells (Figure 2B). Consistent with the observation of cell proliferation on day 1, there were not obvious differences across these conditions. Similarly, no acute toxic effects were observed on the cytoskeleton structures of the cell culture at the 12-h time point (Figure 2C).

**FIGURE 3:**
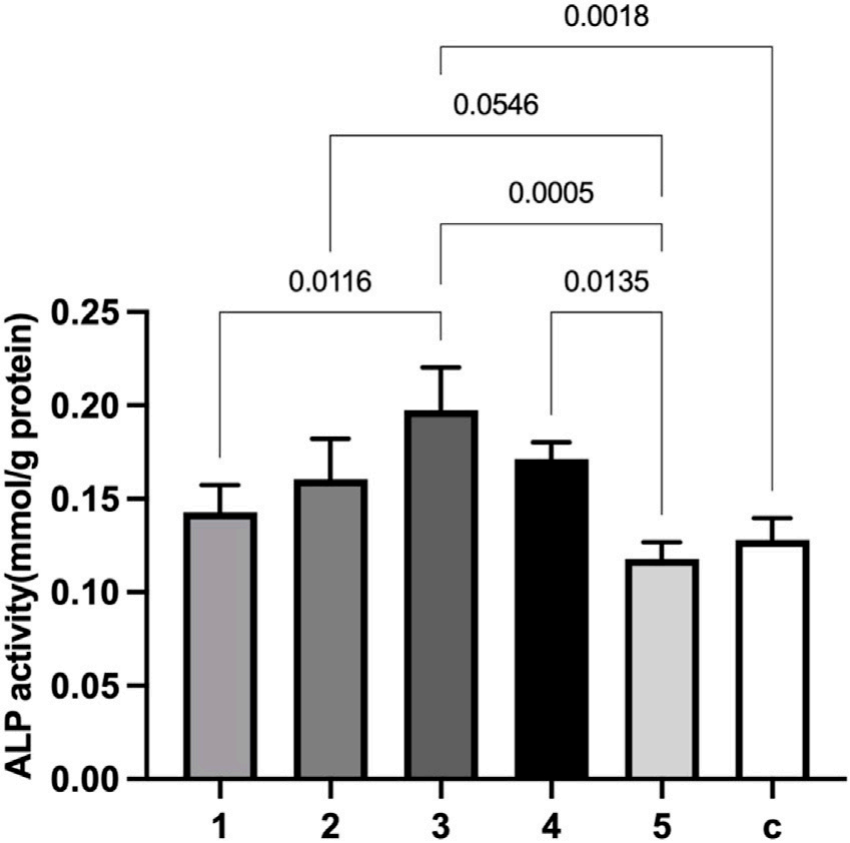
Comparisons of alkaline phosphatase (ALP) activity in MC3T3-E1 culture system after co-incubation with different extract solutions of GDY-Mg alloys. 1 to 5 are GDY-Mg alloys from GDY0.5 to GDY2.5, and c is the control group with the plain medium.

Additionally, alkaline phosphatase (ALP) activity was chosen as an indicator of osteoblast activity due to its direct linkage to the osteoblast phenotype (Mizuno and Kuboki, 2001) and its important role in the skeletal mineralization (Sabokbar et al., 1994). Alloy 3 extract solution significantly enhanced the ALP activity compared to plain medium (*p* = 0.0018). Its effect also statistically differed from those of Alloy 1 (*p* = 0.0116), Alloy 5 (*p* = 0.0005). Extract from Alloy 4 resulted in a more stimulating effect than that from Alloy 5 (*p* = 0.0135) (Figure 3).

### 3.3 Effects on endothelial function (vascularization)

**FIGURE 4:**
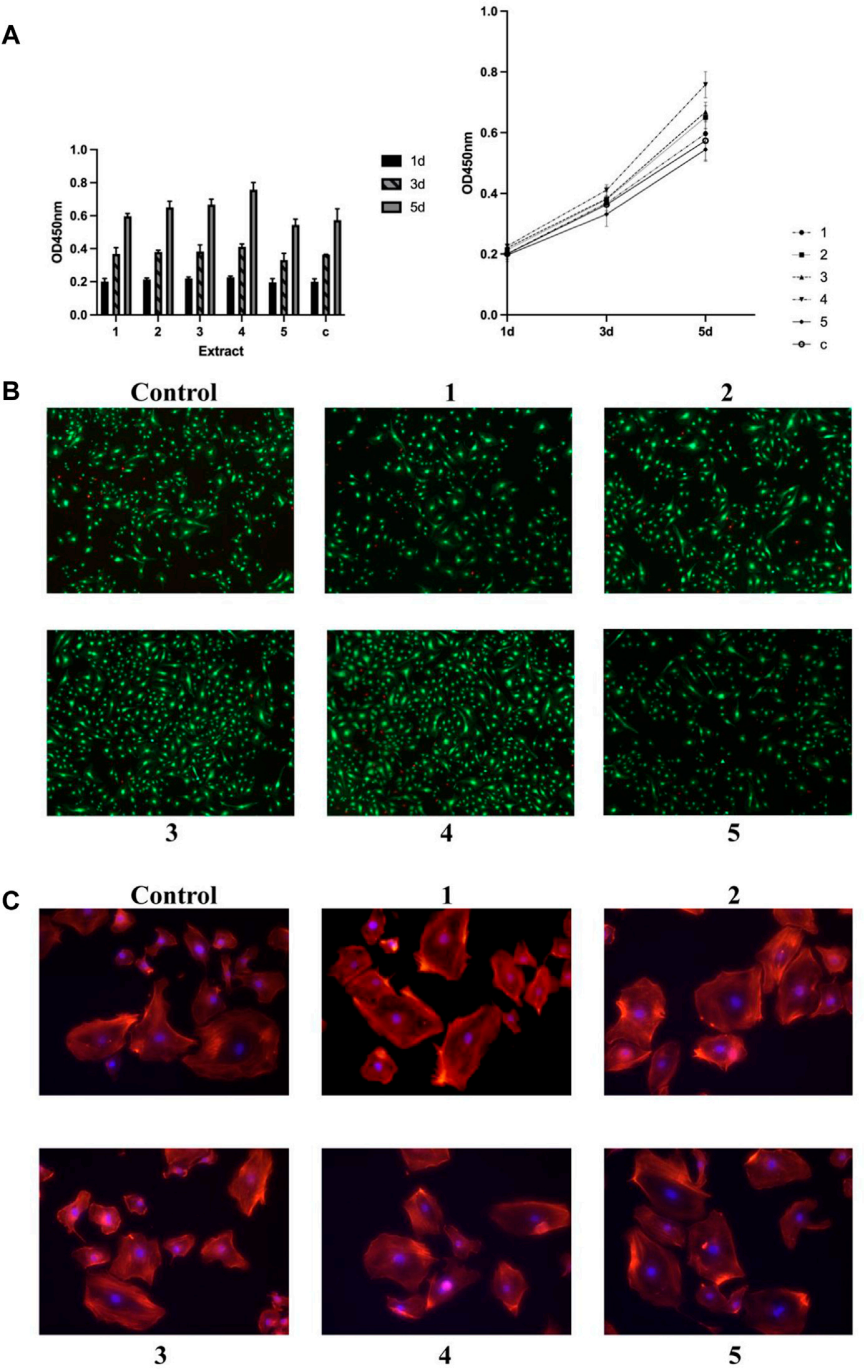
Effects of metal extract solutions from different compositions of GDY-Mg alloys on the viability, proliferation, and cytoskeleton structure of HUVEC cell culture. **(A)** Cell proliferation levels visualized by group (left) and time point (right). **(B)** Live/dead staining. **(C)** Cytoskeleton staining with phalloidin. 1 to 5 are GDY-Mg alloys from GDY0.5 to GDY2.5, and c is the control group with the plain medium.

Similar to what was observed in osteoblastic cell cultures, the cell proliferation, viability, as well as cytoskeleton structures were investigated in endothelial cultures (HUVECs), too (Figure 4).

On day three of incubation, cells treated with the extract solution from Alloy 4 showed a slight but significantly higher proliferation level compared to those that received the extract from Alloy 5 (*p* = 0.0495). Alloy 4 extract solution significantly enhanced the proliferation compared to plain medium (*p* = 0.0017). The effects of extract solution from Alloys 1 and 5 were also significantly less than that from Alloy 4 (*p =* 0.0051 and 0.0005, respectively). Though Alloy 3 extract did not stimulate the proliferation of endothelial cells the most as it did in osteoblast progenitors, it exhibited higher capability than the extract of Alloy 5 (*p* = 0.0324) (Figures 4A, B). Cytoskeleton structures of endothelial cells remained consistent across the groups treated with different alloy extract solutions (Figure 4C).

**FIGURE 5:**
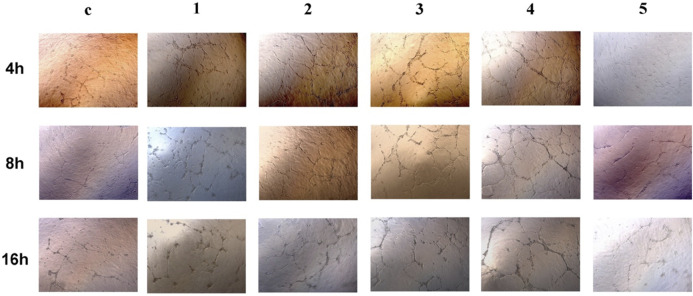
Tube formation of HUVECs at 4 h, 8 h, and 16 h after being seeded onto Matrigel. 1 to 5 are GDY-Mg alloys from GDY0.5 to GDY2.5, and c is the control group with the plain medium.

*In vitro* tube formation assay was done on the matrigel layer to assess the angiogenesis process. Cell cultures treated with extract solution from Alloy 5 exhibited comparable angiogenesis processes with those treated with plain medium (Figure 5). The rest of the metal alloys’ extract solution enhanced the angiogenesis with increased tubes as well as branching sites/nodes (Figure 5).

## 4 Discussion

Magnesium alloys have been extensively studied since the beginning of the 20th century. A medical device made of this promising biomaterial was introduced to the market as early as 2013 (Chen et al., 2014; Seitz et al., 2016). Compared to zinc and iron alloys (Peuster et al., 2001; Li et al., 2008; Schinhammer et al., 2010; Zhang et al., 2010; Vojtěch et al., 2011; Zhang et al., 2012; Bowen et al., 2013), magnesium alloys have many superior benefits: the element itself is a natural and essential component of the human body, with a much higher recommended daily intake level (Lupton et al., 2002). The density and elastic modulus of magnesium is also much closer to the natural bone, which makes the alloys made of it less likely to cause early implant loosening or damage to the healing process (Salahshoor and Guo, 2012). This also assures better results from computed tomography and magnetic resonance imaging with fewer artifacts (Filli et al., 2015).

One of the most well-recognized inevitable characteristics of metals is corrosion. Even though it is most of the time a physiological “homeostasis” in the host body, the frequent execution of various biological reactions still makes it an aggressive environment for the metal alloys. Usually, the corrosion rate can remain at a relatively low level, such as microns each year, to ensure extended usage. The elements in metals or alloys can consistently be released into the surrounding tissue or even the bloodstream. The result can be disastrous to homeostasis. For example, corrosion products of iron have been shown to have threatening effects on the integrity of the arterial wall in animal experiments (Bowen et al., 2012). The goal of exploring beneficial and nontoxic alloying elements is to utilize this idea to achieve more biological benefits from the effects of these additional elements on the functions of host cells/tissue.

However, further applications of magnesium alloys are extensively restricted by their high corrosion rate and bioactivity in physiological environments (Kannan and Raman, 2008). The hydrogen generated along with the biodegrading process of magnesium could result in harmful side effects to the host (Poinern et al., 2012). The introduction of new alloying elements or combinations of several ones can greatly enhance the strength and lower the corrosion rate. On top of these engineering properties of Mg-based alloys, elemental toxicity is another very important factor to consider before the material can be used *in vivo*. Magnesium and rare element-based alloys generally exhibit the highest strength, great ductility, and the best elongation among most of the Mg alloys.

Additional merits of these REEs impacting the biological processes in a positive way attracted more interest recently.

Gadolinium (Gd) is comparatively well-explored in that Gd-based contrast has been used widely in magnetic resonance imaging (Kim et al., 1996). It may cause impaired immune response even though the chances of anaphylaxis decrease at the same time (Farkas and Karácsonyi, 1985; Evans, 1990). The toxicity of Gd compared to other lanthanides is inconsistently observed *in vitro* and *in vivo* (Evans, 1990; Feyerabend et al., 2010). Its genotoxic and mutagenic effects are not well established, either (Yongxing et al., 2000; Webb, 2006; Grillo et al., 2014; Poças et al., 2018). Gadolinium oxide-coated or gadolinium phosphate scaffolds have been confirmed to have promoting effects on fibroblast and bone marrow stem/stromal cells with decent biocompatibility (Zhao et al., 2019; Saranya et al., 2020). Thus, the element Gd has been considered a promising ingredient in the development of bone tissue engineering.

A neighbor of Gd, dysprosium (Dy), also shows acute toxicity when presented as chloride salts or oxide particles (Haley et al., 1966; Evans, 1990), along with moderate mutagenicity (Hasegawa et al., 2012). However, reports on its effect/toxicity as a freely released ion are rather limited. Binary Mg-Dy alloys *in vivo* showed low cytotoxicity, which enabled the further exploitation of Dy as a candidate (Yang et al., 2013). Dysprosium additives provide a substantial increase in strength properties at room and elevated temperatures (Rokhlin and Nikitina, 1999). Higher additions of Dy can enhance the surface morphology features of Mg-alloys (Recktenwald et al., 2020).

The addition of Y can enhance the ductility of magnesium alloys, yet compromising their strength, corrosion resistance, and biocompatibility (Li et al., 2011). But the way how Y is introduced into the matrix also impacts the overall corrosion resistance. The best corrosion resistance can still be achieved with increasing Y concentration as well as proper net Y-rich structure (Liu et al., 2017).

In this study, the effects of these Mg-alloys with different compositions on both endothelial cells and osteoblastic progenitors were preliminarily explored. The increase in the proportions of REEs did not result in significant cell toxicity in either cell line (Figures 1, 3). However, the highest proliferation rates of osteoblastic progenitors and HUVECs were achieved with the presence of extract solution from Alloy 3 and Alloy 4, respectively (Figures 1, 3). Additionally, increased ALP activities were observed when the weight percentages of REEs were moderate (Alloys 2, 3, and 4; Figure 2) as a direct reflection of the execution of the osteoblast function. There were no statistically significant differences among these three compositions, but there was a statistically more apparent positive effect from the extract solution from Alloy 3 compared with the control group (Figure 2). To evaluate the function of the HUVECs, we employed the tube formation assay. Consistent with the results of the proliferation rates, vascularization processes were also enhanced by the extract solutions from Alloys 2, 3, and 4 (Figure 4), suggesting the potential biological benefits of these compositions of Mg-alloys.

Several aspects of the experiments could be advanced in the future with additional validations considering the current limitations of this study. First, caution should be made when extrapolating the results observed from MC3T3-E1 to normal human osteoblasts due to existing differences (Fernandes et al., 2010; Arriero et al., 2012). Even for experiments conducted with human osteoblasts, the differences between the *in vivo* and *in vitro* situations could interfere with the value of the speculations. Second, the methods of detecting the toxicities of REEs could be improved further. In our study, we employed the typical way of making the extraction solutions by allowing the alloys to be immersed in a basal medium. However, this could be insufficient considering the tendencies of REEs to form compounds or participation with constituents of the culture media or physiological fluids (Evans and Evans, 1990). This resulted in divergent ranges of toxicity when interpreting the toxicities of REEs in correlation with their solubilities (Wald, 1989; Pagano et al., 2015). Last but not least, in this study, the exploration of the influences of GDY-Mg alloys on the cytoskeletons of endothelial cells and osteoblasts was restricted to the morphological level. The ALP activities of osteoblastic progenitor cells could also be further validated at the transcriptional and translational levels. These will be focused on in our future studies. If resources permit, we hope to examine the biological functions of GDY-Mg alloys in depth in isolated blood vessels or *in vivo* with animal experiments.

## 5 Conclusion

Overall, this study provided new insights into the biological effects of REE-supplemented magnesium alloys on the viability, proliferation, and function of endothelial and osteoblastic progenitor cells.

1 Increasing weight percentages of Gd-Dy-Y from 0.5% to 0.25% to five-folds did not cause apparent cell death or dysfunction.
2 The moderate amount tested (GDY1.5 and GDY2.0) of REE supplements had the maximal capacity of stimulating the proliferation of both cell types and increasing osteogenesis activity.

The results indicated the great potential of exploiting the bioactivity of complex Mg-alloys, also providing valuable insights into further explorations for optimizing compositions of REEs in novel Mg alloys.

## Data Availability

The raw data supporting the conclusion of this article will be made available by the authors, without undue reservation.
